# Featuring the phenotype of the FMF prototype

**DOI:** 10.1186/1546-0096-13-S1-P78

**Published:** 2015-09-28

**Authors:** I Ben-Zvi, Y Kassel, O Kukuy, C Herskovizh, C Grossman, A Livneh

**Affiliations:** 1 Sheba Medical Center, Internal Medicine F, Ramat-Gan, Israel

## Background

The presentation of FMF is extremely variable, ranging from a quiescent to severe disabling disease. The M694V mutation is one of approximately 300 published genetic variations of MEFV and is thought to be associated with a typical clinical picture of the disease, but studies featuring the phenotype of homozygous M694V phenotype are meager.

## Objectives

To describe the clinical trait of M694V homozygous FMF as compared to the phenotype of FMF with mixed MEFV genotypes.

## Patients and methods

Fifty seven FMF patients homozygous for the M694V genotype were compared to 56 patients carrying other mutations. A questionnaire, including items related to demographic and clinical features was completed for each patient based on interview, physical examination and file notes.

## Results

Compared with the control group, more patients, homozygous for the M694 mutation, suffered from a severe disease (p=0.001), had higher frequency of attacks before and during colchicine treatment (p=0.0001 and 0.0007, respectively), had more related diseases (p=0.0373) and needed higher dose of colchicine to control their disease (p=0.0001). Most other features tested (Table 1) appeared to be more pronounced in M694V homozygous patients (either with or without statistical significance).

## Conclusion

The phenotype of FMF, as manifested in M694V homozygous patients, is the gold standard, to which other FMF presentations should be compared.

**Table 1:** 

Parameter	694 Homozygous (N=57)	Other mutations (N=56)	P
Average length of attack (days)	2.66±1.5	3.03±1.2	0.073

Abdominal attacks	50 (87.7%)	48 (85.7%)	0.788

Arthritis attacks	52 (91.3%)	28 (50%)	**<0.0001**

Pleuritis attacks	36 (46.2%)	18 (38.2%)	**0.0013**

Exertional leg-pain	47 (82.5%)	36 (64.3%)	**0.034**

ELE attacks	10 (17.5%)	3 (5.4%)	0.073

Attacks of fever alone	20 (35.1%)	12 (21.4%)	0.143

Average colchicine dose (mg/day)	1.9±0.48	1.48±0.54	**0.0001**

IV colchicine treatment	5 (8.8%)	0 (0%)	0.057

Proteinuria or amyloidosis	6 (10.5%)	1 (1.8%)	0.113

Anemia of chronic disease	14/53 (26.4%)	7/52 (13.5%)	0.142

Elevated acute phase reactants	10/18 (55.6%)	4/16 (25%)	0.092

Chronic renal failure	6 (10.5%)	0 (0%)	**0.027**

Chronic arthritis	11 (19.3%)	2 (3.6%)	**0.015**

Work days lost each month	4.4±7.2	2.6±4.6	0.718

Harm to quality of life	(1-10) 5.6±3.3	4.1±3	**0.013**

Number of attacks per year w colchicine	7.2±7.8	3.5±5.5	**0.0007**

Number of attacks per year w/o colchicine	23.6±9.3	15.6±11.7	**0.0001**

Crohn's disease	4 (7%)	2 (3.6%)	0.679

Ankylosing Spondylitis	3 (5.3%)	1 (1.8%)	0.619

Behcet's Disease	7 (12.3%)	1 (1.8%)	0.061

Henoch Schonlein Purpura	1 (1.8%)	0	1

All FMF associated diseases	17 (29.8%)	7 (12.5%)	**0.0373**

